# Kidney autotransplantation for the treatment of renal artery occlusion after endovascular aortic repair: a case report

**DOI:** 10.1186/s12882-019-1353-7

**Published:** 2019-05-14

**Authors:** Atsuko Uehara, Tomo Suzuki, Soichiro Hase, Hirofumi Sumi, Satoshi Hachisuka, Eisuke Fujimoto, Kouichirou Aida, Ryuto Nakazawa, Hideo Sasaki, Junki Koike, Tatsuya Chikaraishi, Yugo Shibagaki, Yuhji Marui

**Affiliations:** 1 Division of Nephrology and Hypertension, Department of Internal Medicine, St. Marianna University School of Medicine, Kawasaki, Kanagawa Japan; 2 Department of Nephrology, Kameda Medical Center, Chiba, Japan; 3 Department of Radiology, Kawasaki Saiwai Hospital, Kawasaki, Kanagawa Japan; 4 Department of Nephrology and Hypertension, Kawasaki Municipal Tama Hospital, Kawasaki, Kanagawa Japan; 5 Department of Urology, St. Marianna University School of Medicine, Kawasaki, Kanagawa Japan; 6 Department of Pathology, Kawasaki Municipal Tama Hospital, Kawasaki, Kanagawa Japan

**Keywords:** Acute kidney injury, Collateral circulation, Endovascular aneurysm repair, Kidney autotransplantation, Percutaneous transluminal renal artery angioplasty, Renal artery occlusion

## Abstract

**Background:**

Unintentional renal artery occlusion after endovascular aneurysm repair (EVAR) for abdominal aortic aneurysm remains one of the most unfavorable complications. Renal salvage options include percutaneous transluminal renal artery angioplasty (PTRA) and open hepatosplenorenal bypass. However, the usefulness of kidney autotransplantation (AutoTx) remains unclear.

**Case presentation:**

A 76-year-old woman with a right solitary kidney attributable to a left renal thromboembolism had previously undergone EVAR with a stent graft for an infrarenal aortic aneurysm, which led to ostial occlusion of the right renal artery. In addition, she had undergone PTRA and stenting. Two days before admission, she developed leg edema and hypertension, leading her to visit the hospital. Her serum creatinine level was 2.4 (baseline, 1.0) mg/dL. Acute kidney injury due to renal artery in-stent restenosis was suspected; re-angioplasty was attempted on day 2 of hospitalization, but was unsuccessful. Her renal function did not improve and anuria persisted; thus, hemodialysis was initiated on the same day. The right kidney size (8.6 cm) was preserved relative to her body size, with only mild cortical atrophy. Doppler ultrasonography and mercaptoacetyltriglycine scintigraphy revealed minimal but significant perfusion of the right kidney. Therefore, we considered that kidney perfusion was sustained and renal function could be reversed. On day 25 of hospitalization, right kidney AutoTx to the right iliac fossa was performed to reestablish adequate renal perfusion and reverse the need for dialysis. Soon after the procedure, the patient started passing urine. Her renal function improved; her serum creatinine level decreased to 1.0 mg/dL on day 33 of hospitalization. Hemodialysis was discontinued after the surgery. Zero-hour kidney biopsy showed only mild tubular injury, with neither tubular necrosis nor glomerular abnormalities.

**Conclusions:**

Kidney AutoTx can be performed for patients with renal artery in-stent occlusion after unsuccessful PTRA who previously underwent EVAR. Our case showed successful recovery of renal function nearly 1 month after renal artery occlusion, indicating that revascularization should be considered even if it is delayed, as the kidney might be perfused through collateral circulation.

## Background

Unintentional renal artery occlusion (RAO) after endovascular aneurysm repair (EVAR) for abdominal aortic aneurysm (AAA) remains one of the most unfavorable complications [1]. Renal salvage options include percutaneous transluminal renal artery angioplasty (PTRA) and open hepatosplenorenal bypass [2]. Although PTRA is less invasive, in-stent restenosis remains a frequently encountered problem, reaching 20% at 1 year after PTRA [3]. Open hepatosplenorenal bypass can contribute to long-term bypass patency; however, it is invasive and can cause liver and spleen injury or infarction [4]. In contrast, the usefulness and safety of kidney autotransplantation (AutoTx) as a treatment for renal artery stenosis after PTRA have only been rarely reported [5].

The human kidney can tolerate complete ischemia for approximately 60–90 min at normothermia; therefore, successful recovery of renal function after revascularization that occurs several weeks after RAO was not thought to be possible [6]. Herein, we report on a patient with a solitary kidney who developed dialysis-dependent renal failure due to in-stent restenosis after PTRA for postoperative RAO after EVAR, which was successfully treated with kidney AutoTx 25 days after RAO.

## Case presentation

**Fig. 1:**
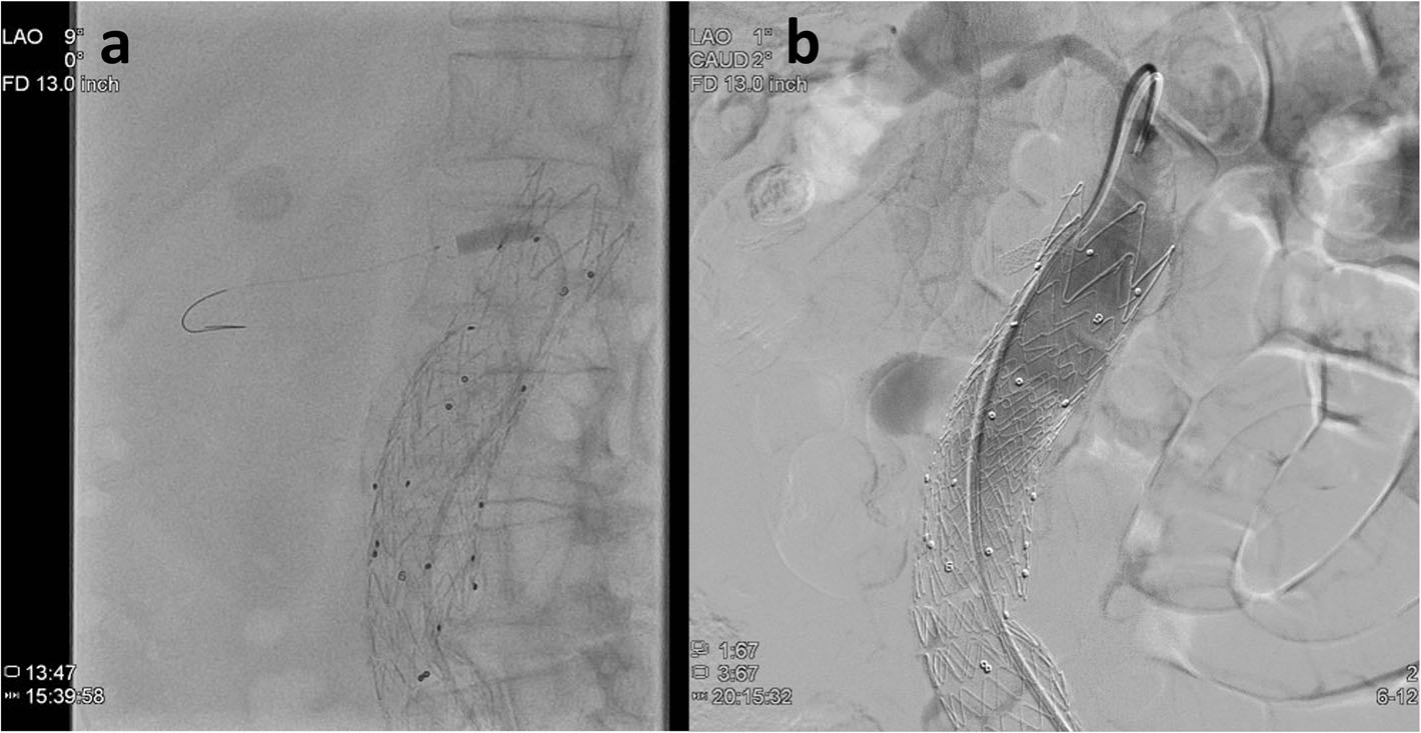
**a** Two years before admission, the patient had undergone successful PTRA as treatment for renal artery occlusion after EVAR. **b** Two years after the first PTRA, she developed anuric AKI due to renal artery in-stent occlusion. PTRA for the right renal artery was attempted; however, it was unsuccessful because the guide wire did not pass through the ostium of the renal artery. PTRA, Percutaneous transluminal renal artery angioplasty; EVAR, Endovascular aneurysm repair; AKI, Acute kidney injury

The patient was a 76-year-old woman with a history of left renal thromboembolism, hypertension, atrial fibrillation, hyperthyroidism, stroke, and AAA. Two years before admission, she had undergone EVAR with a stent graft for infrarenal aortic aneurysm, which led to right ostial RAO. Her left kidney was already atrophic due to previous renal thromboembolism, causing her to develop dialysis-dependent renal failure immediately after EVAR. Twenty-two days after EVAR, she underwent successful PTRA and stenting, and hemodialysis was discontinued (Fig. 1a). However, 2 years after EVAR, she developed acute onset of leg edema, anuria, and hypertension for 2 days, and thus went to the hospital. She was found to have significant worsening of renal function, with a serum creatinine level of 2.4 mg/dL, which had increased from a recent baseline level of 1.0 mg/dL. Acute kidney injury (AKI) due to renal artery in-stent restenosis was suspected, and she was admitted to the hospital. On day 2 of hospitalization, renal artery angiography demonstrated that the right renal artery could not be visualized, thereby confirming the diagnosis of AKI due to right renal artery in-stent occlusion. On the same day, PTRA for the right renal artery was attempted; however, it was unsuccessful as the guide wire could not pass through the ostium of the renal artery (Fig. 1b). Given that her renal function did not improve and anuria persisted, hemodialysis was initiated on the same day. She was transferred to our hospital for preparation of regular hemodialysis on day 9 of hospitalization.

On admission, she had a blood pressure of 202/109 mmHg, heart rate of 76 beats/min, and respiratory rate of 16 breaths/min. During examination, no extremity edema was noted, and no bruit was detected on abdominal auscultation. Her laboratory results at the time of admission were as follows: serum creatinine level, 9.5 mg/dL; estimated glomerular filtration rate (eGFR), 3.5 mL/min/1.73 m^2^; serum albumin level, 3.2 g/dL; serum potassium level, 3.8 mEq/L; serum calcium level, 8.6 mg/dL; serum phosphate level, 4.7 mg/dL; and hemoglobin level, 10.1 g/dL.

**Fig. 2:**
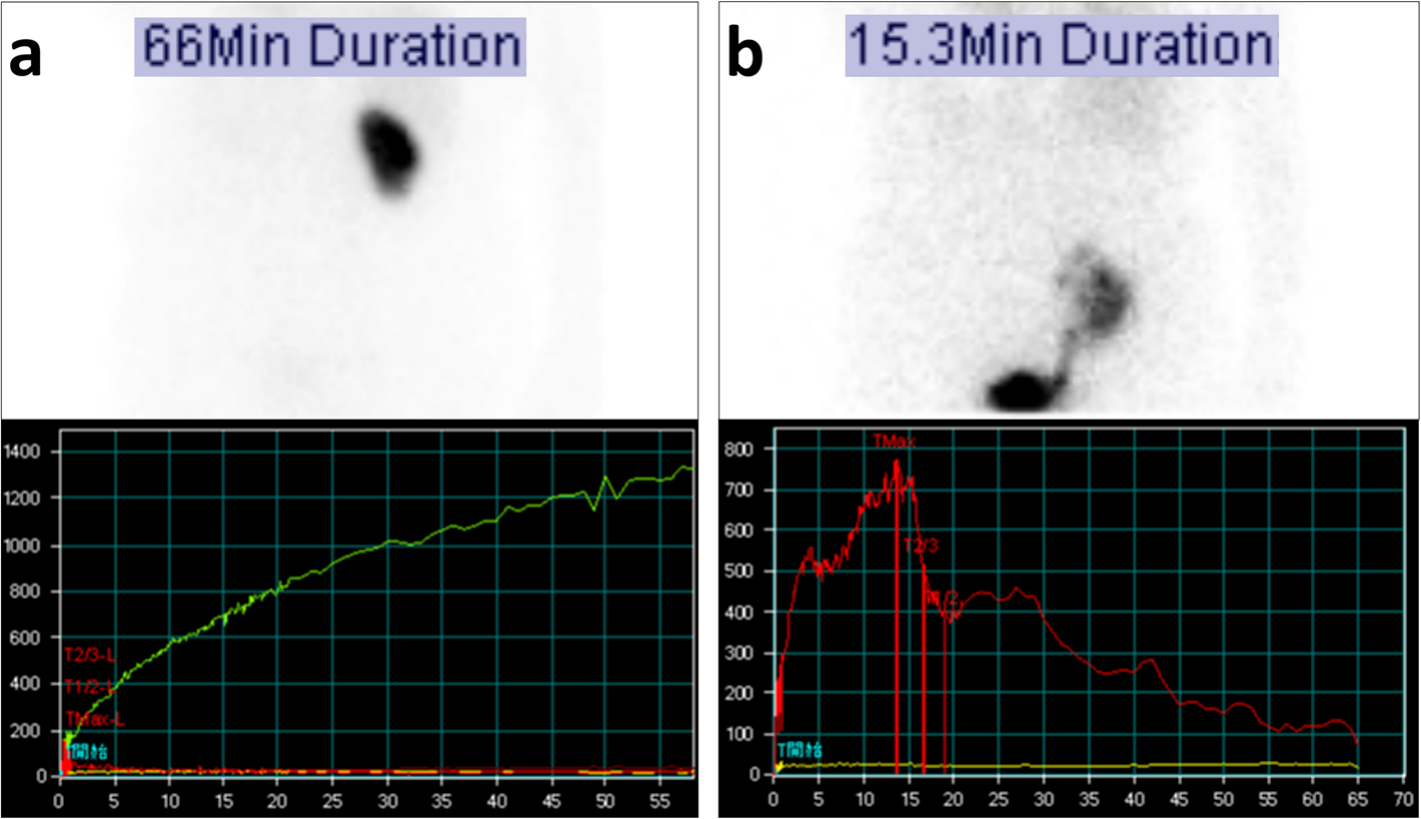
**a** Renal MAG-3 scintigraphy performed on admission demonstrated slow staining of the right kidney; however, MAG-3 did not wash out even at 66 min after injection, indicating that while renal perfusion was sustained, it was not sufficient to sustain GFR, resulting in anuria. **b** A repeat renal MAG-3 scintigraphy performed 4 months after surgery demonstrated smooth staining of the transplanted right kidney at the right iliac fossa; additionally, MAG-3 washed out at 15 min after injection, indicating that renal perfusion and glomerular filtration had recovered. MAG-3, Mercaptoacetyltriglycine; GFR, Glomerular filtration rate

**Fig. 3:**
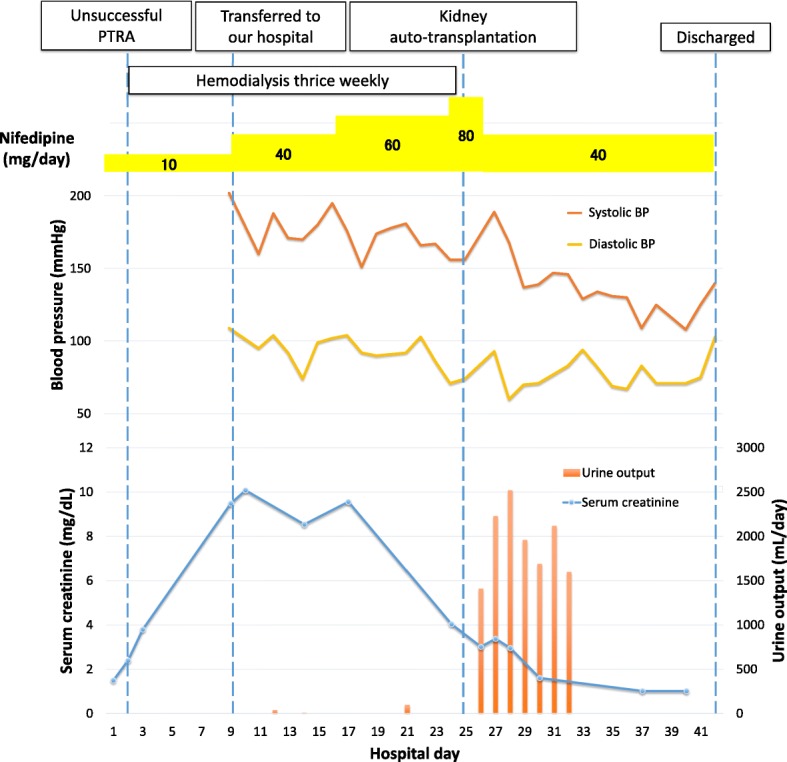
The trends for serum creatinine level, urine output, blood pressure, and dose of nifedipine during hospitalization are shown. The patient started passing urine just after kidney autotransplantation, and hemodialysis was discontinued. Furthermore, refractory hypertension was controlled by a lower dose of nifedipine postoperatively. BP, blood pressure; PTRA, Percutaneous transluminal renal artery angioplasty

**Fig. 4:**
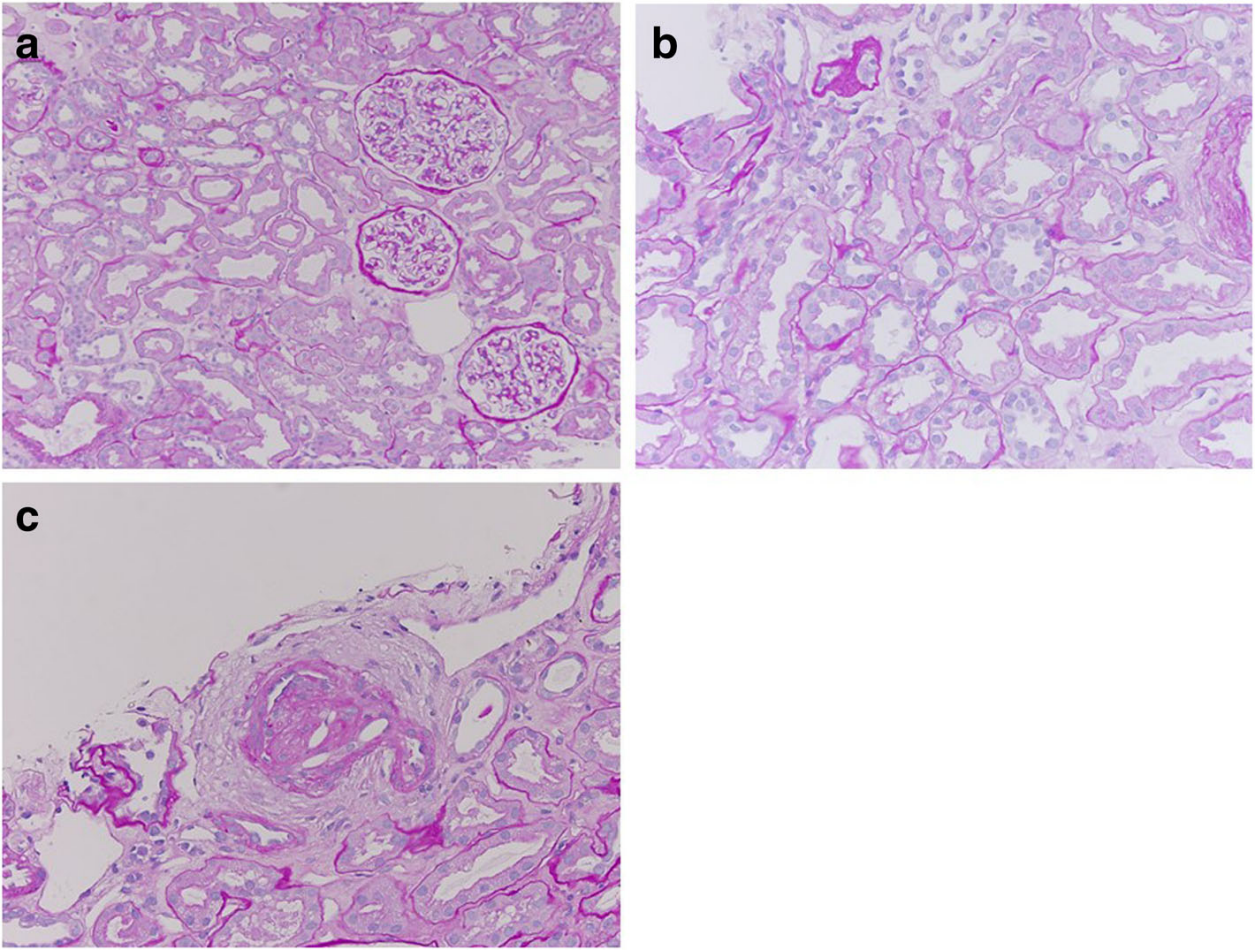
Light microscopy findings for 0-h kidney biopsy performed during kidney autotransplantation. **a** There were no glomerular abnormalities. **b** Mild tubular injury was seen, but there was no tubular necrosis. **c** Cholesterol embolus was observed in one interlobular artery

Anuric AKI persisted after she was transferred to our hospital. In addition to RAO, we suspected contrast-induced nephropathy or cholesterol emboli as a differential diagnosis. However, these were unlikely as she developed AKI before contrast medium was administered and before the intravascular catheter procedure was performed; moreover, there were no signs of peripheral atheroembolism. Her high plasma renin activity (10.7 ng/mL/h, with a plasma aldosterone level of 140 pg/mL), which was tested to determine the cause of hypertension, raised the suspicion that she had a minimally but significantly perfused right kidney. Doppler ultrasonography revealed a renal interlobular artery flow inside the right kidney, indicating that the perfusion of the kidney was sustained, at least at a low level. Renal mercaptoacetyltriglycine (MAG-3) scintigraphy revealed that the right kidney had stained slowly and MAG-3 had not washed out even at 66 min post-injection. The absence of MAG-3 washout meant that the function of the kidney decreased severely, which did not rule out contrast induced AKI, but at least indicated that while renal perfusion was sustained, it was not sufficient to sustain the GFR (Fig. 2a). Although we initially planned to create an arteriovenous fistula for vascular access for permanent hemodialysis, the results of these renal imaging studies led us to reconsider that the AKI may be reversible if the renal artery was revascularized. However, a second attempt of PTRA was considered challenging, given that the renal artery was almost completely occluded. With no prior experience performing hepatorenal bypass, a transplant surgeon on our team considered kidney AutoTx to be a feasible alternative; therefore, kidney AutoTx was performed on day 25 of hospitalization. The renal artery and vein were anastomosed to the external iliac artery and vein, respectively. Cold and warm ischemia time of the right kidney were 84 and 7 min, respectively. After declamping the external iliac artery, the kidney perfused well, except for the upper one-fifth portion, which appeared to be perfused by an upper polar renal artery. Hence, we decided to anastomose the upper polar renal artery to the inferior epigastric artery; this successfully perfused the upper kidney region. Intraoperatively, the patient started passing urine. For the first 24 h postoperatively, her urine output was 2.2 L, and hemodialysis was discontinued (Fig. 3). Her serum creatinine level decreased to 1.02 mg/dL by postoperative day 22, which was almost equivalent to her baseline creatinine level. Her hypertension improved, and the dose of nifedipine was successfully reduced. Her 0-h kidney biopsy during the surgery showed only mild tubular injury and no tubular necrosis. Of 24 glomeruli, 11 showed global glomerular sclerosis, and the remaining 13 exhibited no acute changes. Mild arteriosclerosis with only one cholesterol embolism was observed. Thus, no histological changes were noted that could account for prolonged ischemia leading to anuric AKI (Fig. 4). The patient was discharged on day 42 of hospitalization, and her kidney function remained stable for 2 years, with a creatinine level of 1.03 mg/dL. Four months after surgery, a repeat renal MAG-3 scintigraphy showed that the transplanted right kidney at the right iliac fossa stained smoothly, and MAG-3 washed out 15 min after injection, indicating that renal perfusion and glomerular filtration has recovered (Fig. 2b).

## Discussion and conclusions

In this report, we describe a case with intractable in-stent occlusion of renal artery post-EVAR leading to dialysis-dependent anuric AKI that was successfully treated with kidney AutoTx. To the best of our knowledge, this is the first case in which AutoTx was performed for RAO after EVAR. There are two intriguing clinical messages that we would like to emphasize. First, kidney AutoTx, as well as hepatosplenorenal bypass, can be an effective alternative to PTRA in treating in-stent RAO after EVAR. Second, revascularization should be considered even if dialysis-dependent anuric AKI has persisted for a long duration (nearly 1 month), and even when some extent of renal perfusion appears to be maintained.

Reports on the occurrence of RAO after EVAR have been increasing as EVAR has become widely used for treating infrarenal aortic aneurysms [7]. Given that RAO after EVAR can lead to postoperative dialysis-dependent renal failure when revascularization is not attempted [1], many types of salvage maneuvers for the renal artery, including PTRA or ileorenal, hepatorenal, and splenorenal bypasses, have been attempted. Adu et al. suggested treatment algorithms for unintentional RAO after EVAR. If the RAO is promptly recognized, early treatment strategies should include PTRA, whereas late treatment strategies should include open surgery, as the stent graft deployed by EVAR is likely to be deeply adherent to the aorta, rendering PTRA is more difficult as time passes [2]. However, PTRA is primarily performed based on the accessibility to the renal orifices and, even if the timing is considered late, cases of successful PTRA 4 days after RAO have been reported [8]. Thus, reports on PTRA and bypasses for RAO after EVAR have been increasing [8–10]; however, to the best of our knowledge, our case is the first in which kidney AutoTx was selected as a renal artery salvage option after EVAR. We did not choose to reattempt PTRA because the right renal artery was completely occluded during first PTRA, and the guide wire did not pass through the renal artery orifice. Furthermore, PTRA is more difficult in cases of RAO following EVAR compared with ordinary renal artery stenosis, because the stent graft deployed by EVAR is deeply adherent to the aorta. Additionally, there are several reasons why we decided to perform kidney AutoTx and not bypass surgery. Our surgeons had more expertise in kidney transplantation than bypass surgery. In addition, an advantage of kidney AutoTx over bypass is that the warm ischemic time can be reduced to a few minutes as the kidney is preserved under cold conditions, which is optimal in the preservation of renal function [11]. However, kidney AutoTx might not be an appropriate option if severe iliac arterial atherosclerosis is noted, as such patients can develop renal artery anastomosis failure or acute limb ischemia due to the steal phenomenon [12]. In our case, we did not check ankle-brachial index to rule out severe atherosclerotic external iliac artery disease prior to surgery. However, the patient denied intermittent claudication and dorsal arteries of foot were palpable and the CT scan revealed only mild calcification of external iliac arteries. Given the information provided, we concluded that peripheral artery disease was unlikely. The warm ischemic time during bypass surgery is also < 30 min (albeit longer than that during kidney AutoTx), which does not lead to renal ischemia; however, caution is required when performing bypass surgeries. If the celiac artery is not patent, hepatorenal bypass and splenorenal bypass are not suitable as they can lead to infarction of the liver and spleen, respectively [2]. Furthermore, hepatorenal bypass is contraindicated when the patient has liver dysfunction, and splenorenal bypass requires a great exposure and is associated with a risk of splenic and pancreatic injuries [4]. Both ileorenal bypass and kidney AutoTx are not suitable when severe iliac arterial atherosclerosis is present. In our case, the celiac artery was patent and iliac arterial atherosclerosis was mild; therefore, a hepatorenal or ileorenal bypass could have been performed.

In the present case, we believed that the kidney became ischemic because of the one-month duration of hypoperfusion. We were also unsure whether the kidney function would be reversible after kidney AutoTx. However, postoperatively, the patient quickly achieved almost full recovery of right renal function, which exceeded our expectations. More surprisingly, in the 0-h kidney biopsy specimen, neither tubular necrosis nor glomerular abnormalities, which would be expected in cases of RAO, were noted, even though dialysis-dependent anuric AKI persisted for nearly 1 month. We believe that this is the first case report with renal histological findings in the case indicating prolonged anuric AKI requiring dialysis. Human kidneys can tolerate complete ischemia for approximately 60–90 min at normothermia, and successful recovery of renal function after revascularization several weeks after RAO was not thought to be possible [6]. Revascularization via bypass surgery 6 days after RAO was considered “delayed” in another published case report [10]. Our case showed successful recovery of renal function nearly 1 month after RAO. The reason that this patient’s kidney was able to tolerate ischemia for such a prolonged period of occlusion may be explained by the presence of collateral vessels that maintained kidney viability, which allowed the significant perfusion to significant stenosis [13]. The kidneys receive collateral blood supply from the aorta, lumbar, periureteral, peripelvic, and adrenal vessels after RAO [14]. In our patient, anastomosing the polar artery to the inferior epigastric artery led to the kidney becoming well-perfused, implying that the polar artery originating directly from the aorta was one of the collateral vessels. The 0-h biopsy was taken from the kidney ex-vivo on the side table by the operation bed, and we took the specimen from the middle portion of the kidney, which could still be supplied by collateral when main artery was occluded. Sampling bias cannot be ruled out completely since we did not check the whole kidney. However, the sample volume of the present case was more sufficient than that of usual percutaneous biopsy, which made sampling bias much less likely. Two years ago, at the time of RAO, our patient’s kidney had tolerated 22 days after EVAR until successful PTRA, implying that the collateral artery also helped maintain perfusion to the kidney at that time. Therefore, it is possible that kidney tissue may remain viable and/or in hibernation, even when there is severely compromised perfusion from the main renal artery, no filtration, and the patient is completely anuric [15].

Several circumstances were unique to our case; remote ischemic preconditioning of right kidney 2 years prior, collateral blood flow to the kidney detected in Doppler ultrasonography or scintigraphy, presence of significant stenosis indicated by high blood pressure and high renin levels, acute onset of anuric AKI, and possible contrast induced AKI. However, if there are collateral arteries to perfuse the kidney, even if the flow is minimal, revascularization should be attempted, even in cases with as long as a 1 month of dialysis dependence. Although identifying collateral vessels is difficult, collateral circulation to the kidney can be detected based on ultrasonography, computed tomography, or other radiologic findings. Renal size and cortical enhancement should be assessed, although they may not be sufficient in determining whether renal function will return to baseline after revascularization [2]. In contrast, the rich collateral circulation detected on angiography was previously reported to be a predictive determinant of kidney salvageability [16]. However, collateral vessels were not observed on angiography in our case, although it is possible that the amount of contrast media used during angiography was too small to detect the distant collateral artery. Doppler ultrasonography is also useful for detecting perirenal circulation [17], and in fact, intrarenal blood flow was detected in our patient. Moreover, results of Doppler ultrasonography and renal scintigraphy prompted us to believe that the kidney dysfunction was reversible if a renal artery revascularization procedure was performed. To our knowledge, no previous reports have assessed the usefulness of renal scintigraphy for evaluating kidney viability.

Our case indicates that even cases with prolonged anuric AKI due to renal artery occlusion may be recovered by kidney AutoTx if kidney perfusion seems to be maintained by collateral vessels, although several circumstances unique to our case may have contributed to the prolonged viability of the ischemic kidney. These collateral vessels can allow the significant perfusion to significant stenosis and can be detected by Doppler ultrasonography or scintigraphy, as well as angiography. As a revascularization method, kidney AutoTx can be a viable option, as well as PTRA and hepatosplenorenal bypass, especially when PTRA is difficult or unsuccessful, and the available surgeons have prior experience of kidney transplant surgery.

## Abbreviations

AAA: Abdominal aortic aneurysm
AKI: Acute kidney injury
AutoTx: Autotransplantation
EVAR: Endovascular aneurysm repair
GFR: Glomerular filtration rate
MAG-3: Mercaptoacetyltriglycine
PTRA: Percutaneous transluminal renal artery angioplasty
RAO: Renal artery occlusion
